# Bibliometric and visualization analysis of radiation brain injury from 2003 to 2023

**DOI:** 10.3389/fneur.2023.1275836

**Published:** 2024-01-08

**Authors:** Baofang Wu, Shaojie Li, Jian Wang, Jiayin Wang, Weizhi Qiu, Hongzhi Gao

**Affiliations:** ^1^Department of Neurosurgery, The Second Affiliated Hospital of Fujian Medical University, Quanzhou, China; ^2^Department of Neurosurgery, The Second Affiliated Clinical Medical College of Fujian Medical University, Quanzhou, China; ^3^Department of Pathology, The Second Affiliated Hospital of Fujian Medical University, Quanzhou, China

**Keywords:** bibliometric analysis, development trends, hot topics, CiteSpace, VOSviewer, R-bibliometrix, radiation brain injury

## Abstract

**Background:**

Over the past two decades, the field of radiation brain injury has attracted the attention of an increasing number of brain scientists, particularly in the areas of molecular pathology and therapeutic approaches. Characterizing global collaboration networks and mapping development trends over the past 20 years is essential.

**Objective:**

The aim of this paper is to examine significant issues and future directions while shedding light on collaboration and research status in the field of radiation brain injury.

**Methods:**

Bibliometric studies were performed using CiteSpaceR-bibliometrix and VOSviewer software on papers regarding radiation brain injury that were published before November 2023 in the Web of Science Core Collection.

**Results:**

In the final analysis, we found 4,913 records written in 1,219 publications by 21,529 authors from 5,007 institutions in 75 countries. There was a noticeable increase in publications in 2014 and 2021. The majority of records listed were produced by China, the United States, and other high-income countries. The largest nodes in each cluster of the collaboration network were Sun Yat-sen University, University of California–San Francisco, and the University of Toronto. Galldiks N, Barnett GH, Langen KJ and Kim JH are known to be core authors in the field. The top 3 keywords in that time frame are radiation, radiation necrosis, and radiation-therapy.

**Conclusions:**

The objective and thorough bibliometric analysis also identifies current research hotspots and potential future paths, providing a retrospective perspective on RBI and offering useful advice to researchers choosing research topics. Future development directions include the integration of multi-omics methodologies and novel imaging techniques to improve RBI's diagnostic effectiveness and the search for new therapeutic targets.

## 1 Introduction

The rapid advancement of nuclear science and technology has led to the widespread use of radioisotopes in various industries, including electric power generation, agriculture, national defense, aerospace, and medical treatment. While the use of radioisotopes has greatly accelerated social progress and brought significant economic benefits, it also comes with some risks due to its unique physical and chemical characteristics. The most devastating nuclear power plant accidents in history occurred at Chernobyl and Fukushima, where hundreds of thousands of people were exposed to radiation. The long-term implications of these incidents are still unknown. In the medical field, Medical isotopes are radioactive substances that are injected or consumed by patients for radiation treatment or imaging diagnostics (nuclear medicine). High-energy rays or particle beams produced by several kinds of X-ray treatment devices or gas pedals are also typically used in radiation therapy. While radiotherapy is a useful therapeutic option for many cancers, it can also cause radiation damage to healthy cells and tissues surrounding the tumor. Clinical investigations have shown that the skin, intestinal, brain, lung, liver, and cardiovascular systems are frequently affected by radiation damage in humans (1). Previous research has shown that radiation treatment can result in significant brain damage even while successfully preventing the formation of CNS tumors (2). In the United States, over 200,000 patients with brain tumors receive treatment using whole brain or partial large field radiation therapy each year (3), and up to 90% of adult brain tumor patients continue to live after radiation therapy for more than 6 months (4). The incidence of radiation brain injury (RBI) is rising as a result of more effective radiation therapy, longer life expectancy, improved and standardized imaging, and other factors (5) that have a positive impact on patient survival (6). In long-term survivors with metastatic brain tumors treated with gamma knife radiosurgery, 64% of patients developed radiation necrosis, with median survival of 32 months (7, 8). Radiation-induced cognitive impairment, including dementia, is reported to occur in up to 50–90% of adult brain tumor patients who survive >6 months post-irradiation (4, 9, 10).

**Figure 1:**
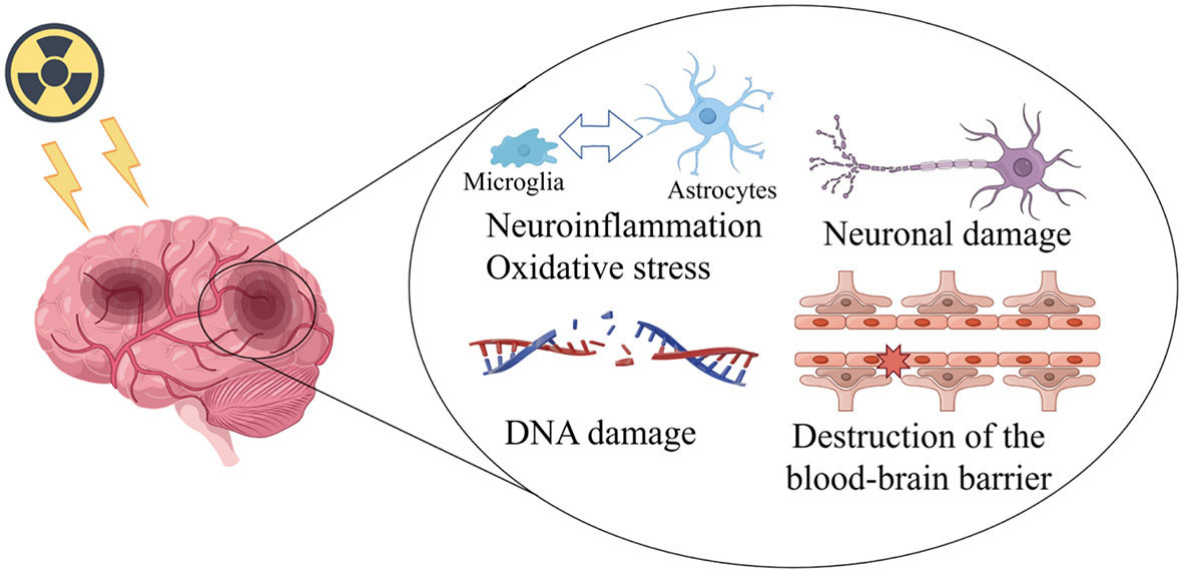
Mechanisms of radiation damage to the brain (This figure was drawn in Figdraw. ID:SSIOW8d79f).

RBI can be classified into three phases based on the period of incidence and clinical presentation (11): acute, early delayed injury, and late delayed injury. Edema, headaches, and drowsiness are symptoms of acute brain injury, which happens days or weeks after irradiation. Drowsiness, focus issues, and exhaustion are signs of early delayed harm that show up 1–6 months after radiation exposure. Early injuries can result in serious bodily reactions, but these reactions are typically brief, reversible, subdued by symptomatic therapy, or even self-repaired by the body. However, late-stage radiation-induced brain damage is progressive and irreversible, and it often manifests 6 months after irradiation. Neurocognitive impairments are caused by vascular abnormalities, demyelination, and white matter necrosis. This cognitive impairment is characterized by decreases in verbal memory, learning and spatial memory, attention, and new problem-solving skills. Multiple hypotheses have been put forth to explain the pathophysiology of RBI, including direct radiation-induced damage (12), the immune inflammatory response (13), oxidative stress (14), and damage to the cerebrovascular system (15). RBI is considered to be a dynamic and complex cascading process (Figure 1). In response to these pathophysiological mechanisms, various research teams have reported on ways to improve the efficacy of radiotherapy or to reduce the toxicity induced by ionizing radiation. These include the use of thalidomide to treat post-radiation cerebral small vessel injury and cognitive dysfunction (16); the use of mesenchymal stem cells to reduce oxidative stress and modulate the inflammatory response (17); and the use of advanced radiation therapy techniques such as stereotactic radiosurgery, stereotactic radiotherapy, intensity-modulated radiation therapy or image-guided radiation therapy to deliver a higher radiation dose to the tumor without increasing the radiation dose to surrounding normal organs (18).

Over the past two decades, the field of RBI has garnered increasing attention from brain scientists, particularly in the areas of molecular pathology and therapeutic approaches. There has been a corresponding increase in publications on this topic. As a result of the rise in publications, academics are now devoting more time and energy to examining the frontiers and research hotspots in their subjects. Furthermore, it might be difficult to adequately describe and summarize these domains' boundaries and research hotspots. A unique, user-friendly, and fascinating method for condensing the copious amounts of data found in papers on radiation brain damage is bibliometrics. Bibliometrics, as defined by Alan Pritchard in 1969, involves the application of mathematical and statistical methods to books and other media of communication (19). This field offers the benefits of visualization, quantification, and knowledge discovery, enabling the creation of a comprehensive picture of a given subject area. Bibliometrics can be used to describe and assess the historical process, current research status, and trends within a subject area (20). For medical researchers, bibliometrics holds significant promise. With the development of digital technology and the growth in the volume of literature, bibliometrics has enabled researchers to stay up to date on new information regarding disciplinary dynamics, drug treatments, diseases, and other health science trends in the field of medicine.

It is helpful to assess the quantitative and qualitative worth of these works from a scientific standpoint in order to further progress research in the field of RBI. For individuals involved in RBI research, this study offers a thorough and in-depth understanding of the knowledge structure and evolution of this multidisciplinary subject through the use of the scientific mapping tool CiteSpace and the R programming language.

## 2 Materials and methods

### 2.1 Data collection

The following search algorithm was used to search the literature available in the Science Citation Index Expanded (SCI-EXPAND-ED) in the Web of Science Core Collection (WoSCC) database since 2003 using the medical subject phrases Radiation and Brain Injury. Search strategy: Edema, Brain OR Intracranial Edema OR Edema, Intracranial OR Brain Swelling OR Brain Swellings OR Swelling, Brain OR Vasogenic Cerebral Edema OR Cerebral Edemas, Vasogenic OR Edema, Vasogenic Cerebral OR Cerebral Edema, Vasogenic OR Cytotoxic Cerebral Edema OR Cerebral Edema, Cytotoxic OR Edema, Cytotoxic Cerebral OR Vasogenic Brain Edema OR Brain Edema, Vasogenic OR Edema, Vasogenic Brain OR Cerebral Edema OR Edema, Cerebral OR Cytotoxic Brain Edema OR Brain Edema, Cytotoxic OR Edema, Cytotoxic Brain OR brain necrosis) OR (Injuries, Brain OR Brain Injury OR Injury, Brain OR Injuries, Acute Brain OR Acute Brain Injuries OR Acute Brain Injury OR Brain Injury, Acute OR Injury, Acute Brain OR Brain Injuries, Acute OR Brain Lacerations OR Brain Laceration OR Laceration, Brain OR Lacerations, Brain OR Brain Injuries, Focal OR Brain Injury, Focal OR Focal Brain Injury OR Injuries, Focal Brain OR Injury, Focal Brain OR Focal Brain Injuries) AND (Radiation OR Radiations) OR (Radiation encephalopathy). Article and review was search type allowed, and English was the only publication language. The total number of documents in the search results was 4,913, and the search results were exported to “Plain Text Format” and “End Note Desktop” and the record content was “Full Record and Cited References”, respectively. The results were captured as a “full record with references cited” and exported to “plain text format” and “End Note Desktop” for bibliometric analysis. All downloaded data was independently checked by two researchers (Jiaying Wang and Baofang Wu). The detailed data retrieval strategies and inclusion criteria for this study are summarized in articles from the WoSCC database and the inclusion criteria for the study.

### 2.2 Data analysis

Microsoft Office Excel 2020 (Microsoft, Redmond, WA, USA) was used for the descriptive statistical analysis and for generating graphs. Meanwhile, a polynomial regression model was used to analyze the trends of annual citations and publications through Microsoft Office Excel 2020. Bibliometric visualization was performed by VOSviewer and CiteSpace V. VOSviewer (Version 1.6.16) is a widely used software in bibliometrics developed by van Eck and Waltman (1). In this study, VOSviewer was used to perform the co-citation analysis of references/journals, co-occurrence analysis of author keywords, and co-authorship analysis of countries/institutions/authors. CiteSpace V (Version 5.8.R3) is also a popular visual tool (21, 22) for co-authorship analysis of institutions, citation burst analysis of keywords, and timeline view analysis of co-cited references in this study. The parameter settings for CiteSpace were as follows: timespan = 2003–2023, slice length = 1, selection criteria = top 50 perslice, node types = (reference, institution, keyword), pruning = (minimum spanning tree, pruning sliced networks), and visualization = cluster view-static.

## 3 Result

**Figure 2:**
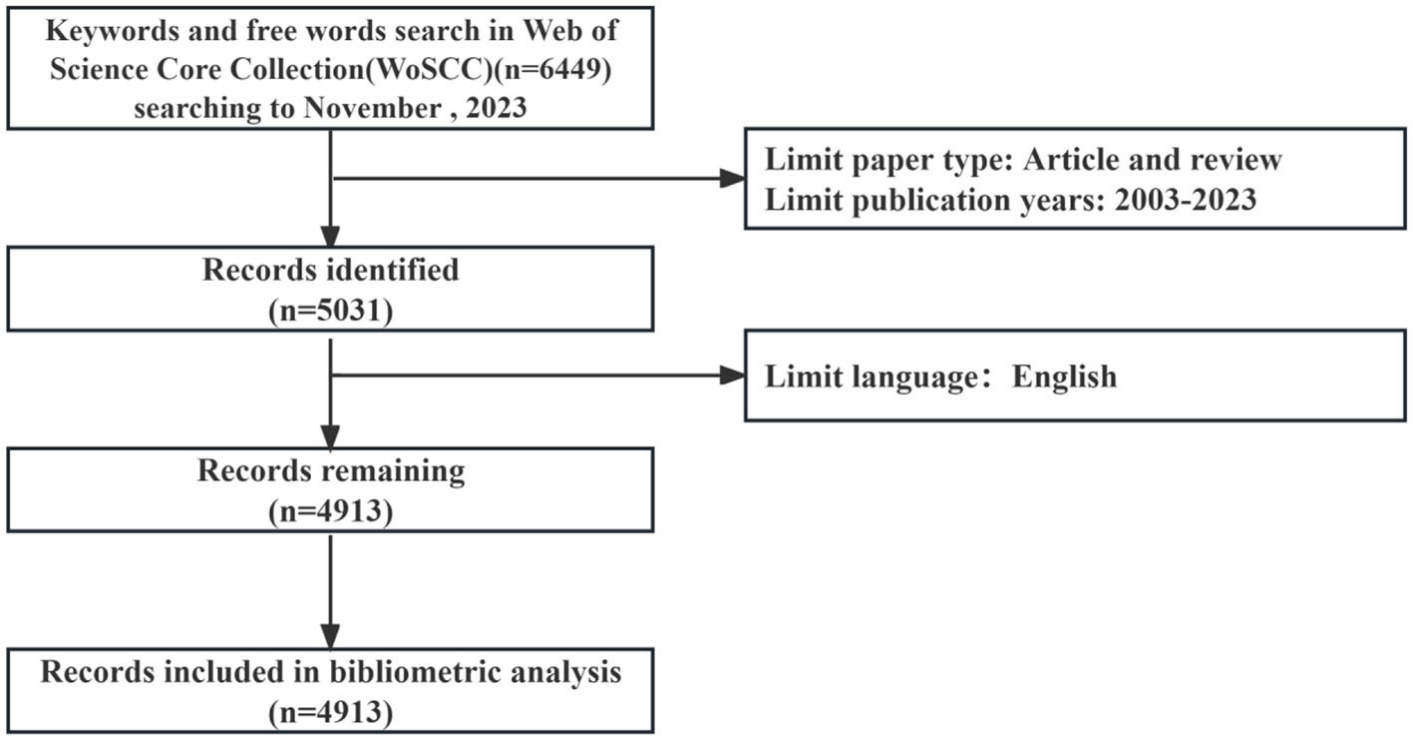
A flowchart representing retrieval strategies for RBI.

**Figure 3:**
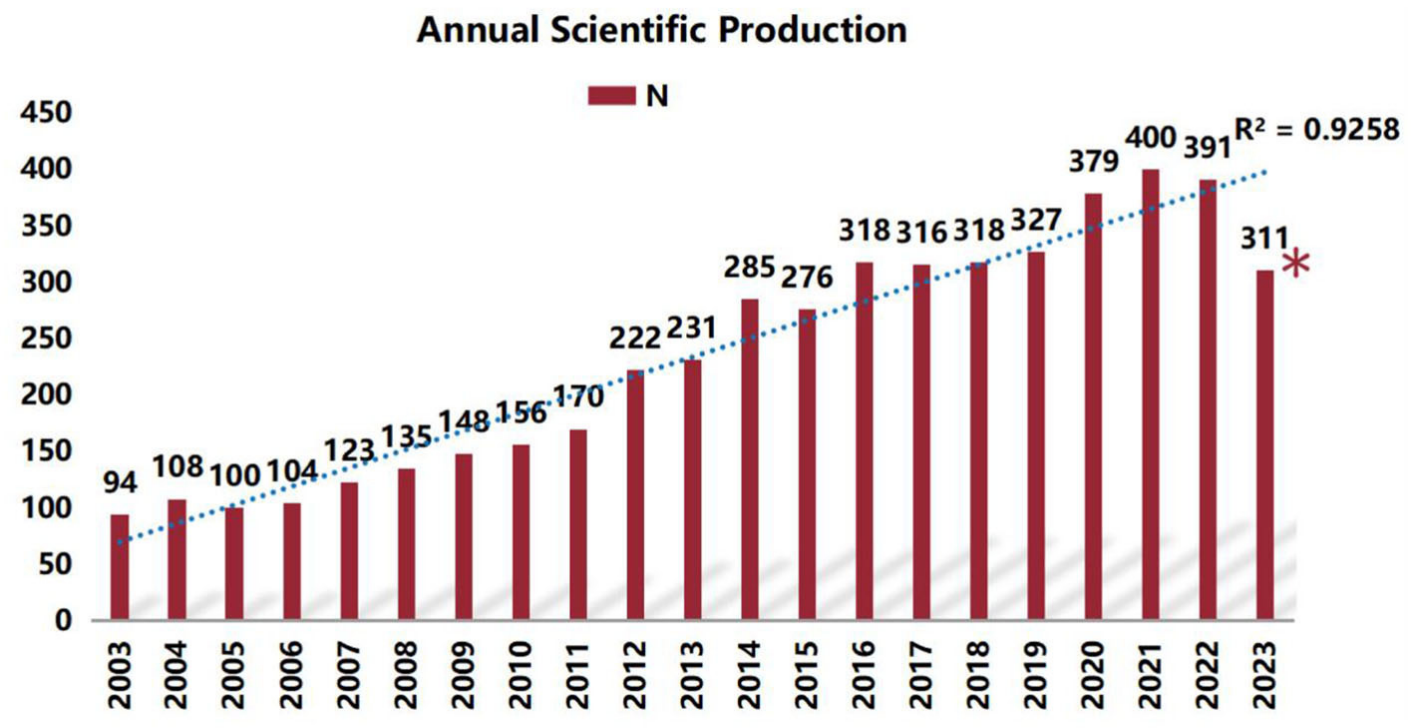
Annual frequency of publications in the field of RBI. (*The number of publications in the first 10 months of 2023).

A total of 4,913 radiation encephalopathy papers were identified, published between January, 2003 and November, 2023 (Figure 2). As shown in Figure 3, the annual output of radiation encephalopathy-related studies exhibits an upward trend from 2003 to 2023. The maximum number of papers were published in 2021 (*n* = 400, 8.1%), with an average annual output of about 245. Since 2016, over 300 papers have been published annually. Figure 3 also shows a strong correlation [coefficient of determination (*R*^2^) = 0.9258] between the publication year and the number of studies through linear fitting of radiation encephalopathy-related studies.

### 3.1 Analysis of countries and districts

**Figure 4:**
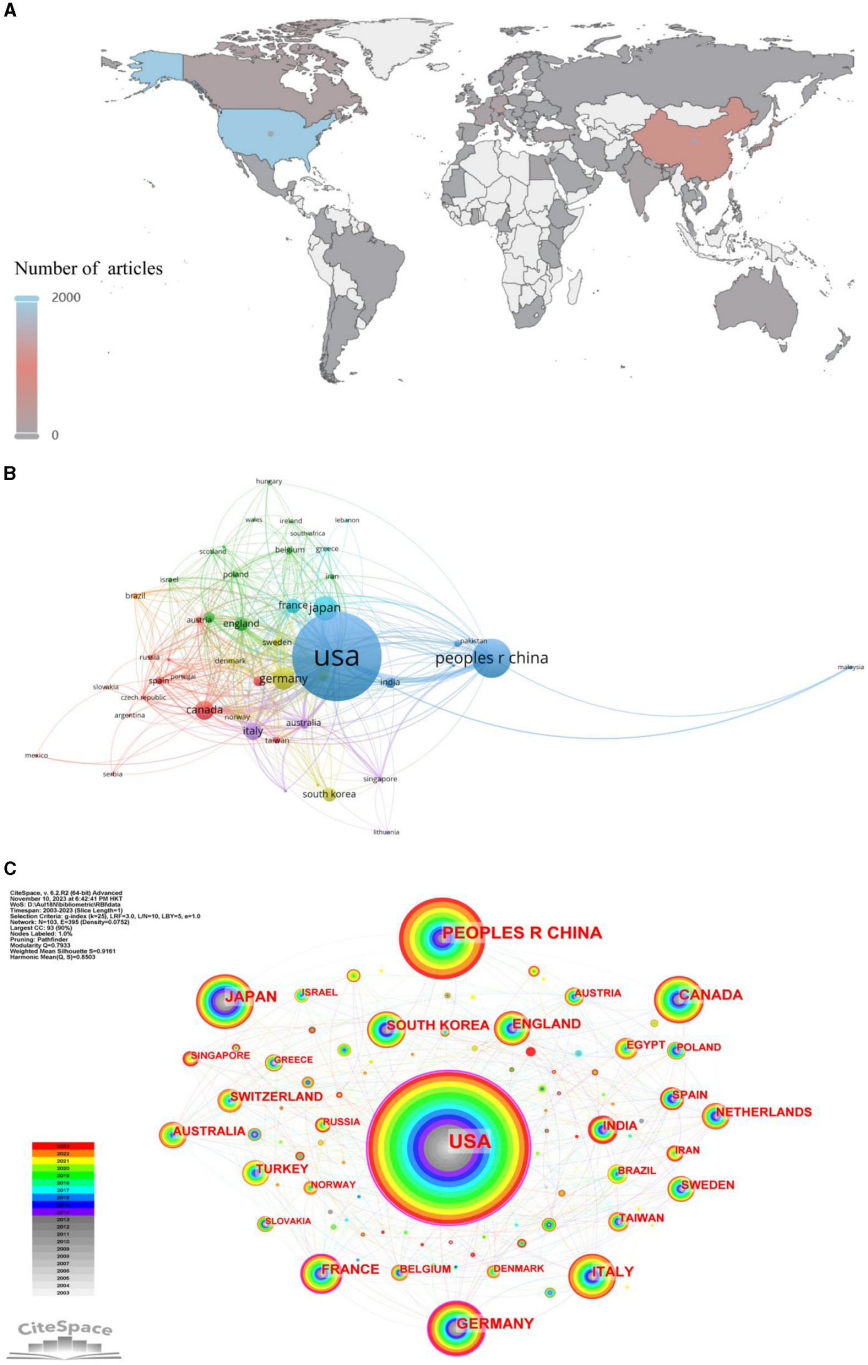
The bibliometric analysis of country on RBI. **(A)** Distribution of articles by country. **(B)** Countries and regions cooperation network. **(C)** Dynamics and trends of countries/regions over years.

A total of 75 countries contributed to publications on radiation encephalopathy worldwide (Figure 4A). Four countries published more than 200 records, with the United States publishing the most (1,961/4,913, 39.9%). The next three most prolific countries were China (672/4,913, 13.7%), Japan (334/4,913, 6.8%), and Germany (106/2,641, 5.0%). The United States also had the highest average number of citations (38.8), followed by France (34.2), Italy (31.2), Germany (29.9), Japan and Canada (24.5) (Table 1). A network map of the top 50 most prolific countries, forming seven main clusters (Figure 4B), showed active collaborations among these countries, particularly between the United States, China, and Canada. From 2021 to 2023 (Figure 4C), the USA, China, Germany, Canada, Japan, Australia, and England conducted more studies in this field and after 2021, many other investigators worldwide began to pay more attention to this field.

**Table 1:** Publication metrics of articles on RBI of the top ten countries by number of publications.

**Rank**	**Country**	**Articles**	**Average article citations**	**Citations**
1	USA	1,961	38.8	76,158
2	China	672	13.7	9,211
3	Japan	334	24.5	8,195
4	Germany	244	29.9	7,302
5	Canada	166	24.5	4,059
6	Italy	162	31.2	5,050
7	Korea	157	19.5	3,057
8	France	130	34.2	4,446
9	Turkey	97	15.1	1,468
10	India	95	13.8	1,307

### 3.2 Analysis of universities and institutions

**Figure 5:**
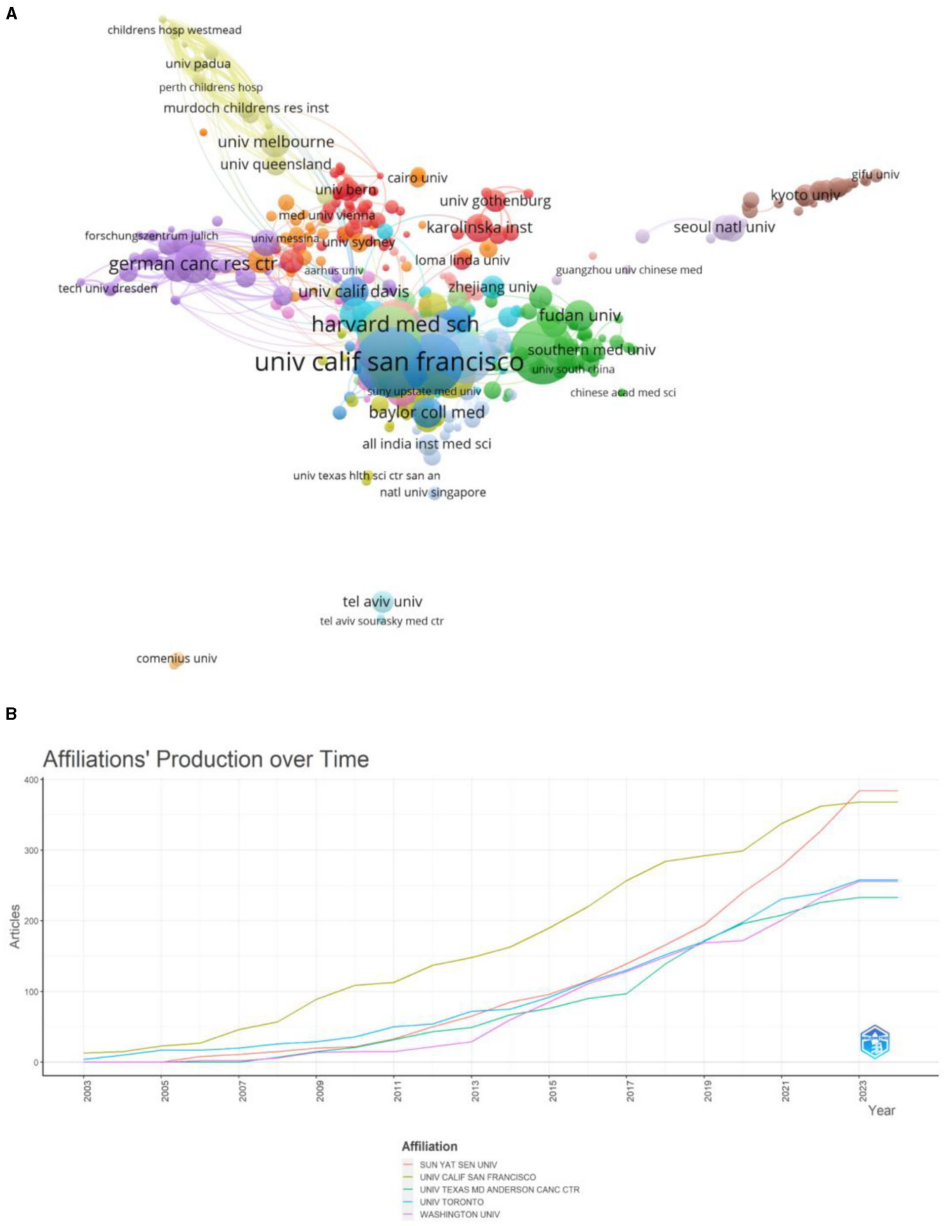
The bibliometric analysis of universities and institutions on RBI. **(A)** The visualization of institutions on research of RBI. **(B)** Affiliations' production over time.

A total of 5,007 universities or institutions contributed to radiation encephalopathy research, and an extensive cooperation network analysis was conducted among these universities or institutions. A network map and overlay visualization of the top 500 institutions by frequency formed 16 clusters based on authors' keywords (Figure 5A). The largest contributors were Sun Yat-sen University, University of California–San Francisco, and the University of Toronto. Cluster 1 was the largest cluster, containing 58 nodes representing different universities or institutions, while Cluster 16 was the smallest with 2 nodes. The results also showed that Sun Yat Sen University, the University of Toronto, Washington University, the University of California San Francisco, and The University of Texas MD Anderson Cancer Center were among the first to contribute to primary and basic research on these topics in 2003. Since then, these studies have gradually gained popularity among universities and institutes (Figure 5B).

### 3.3 Analysis of authors

**Figure 6:**
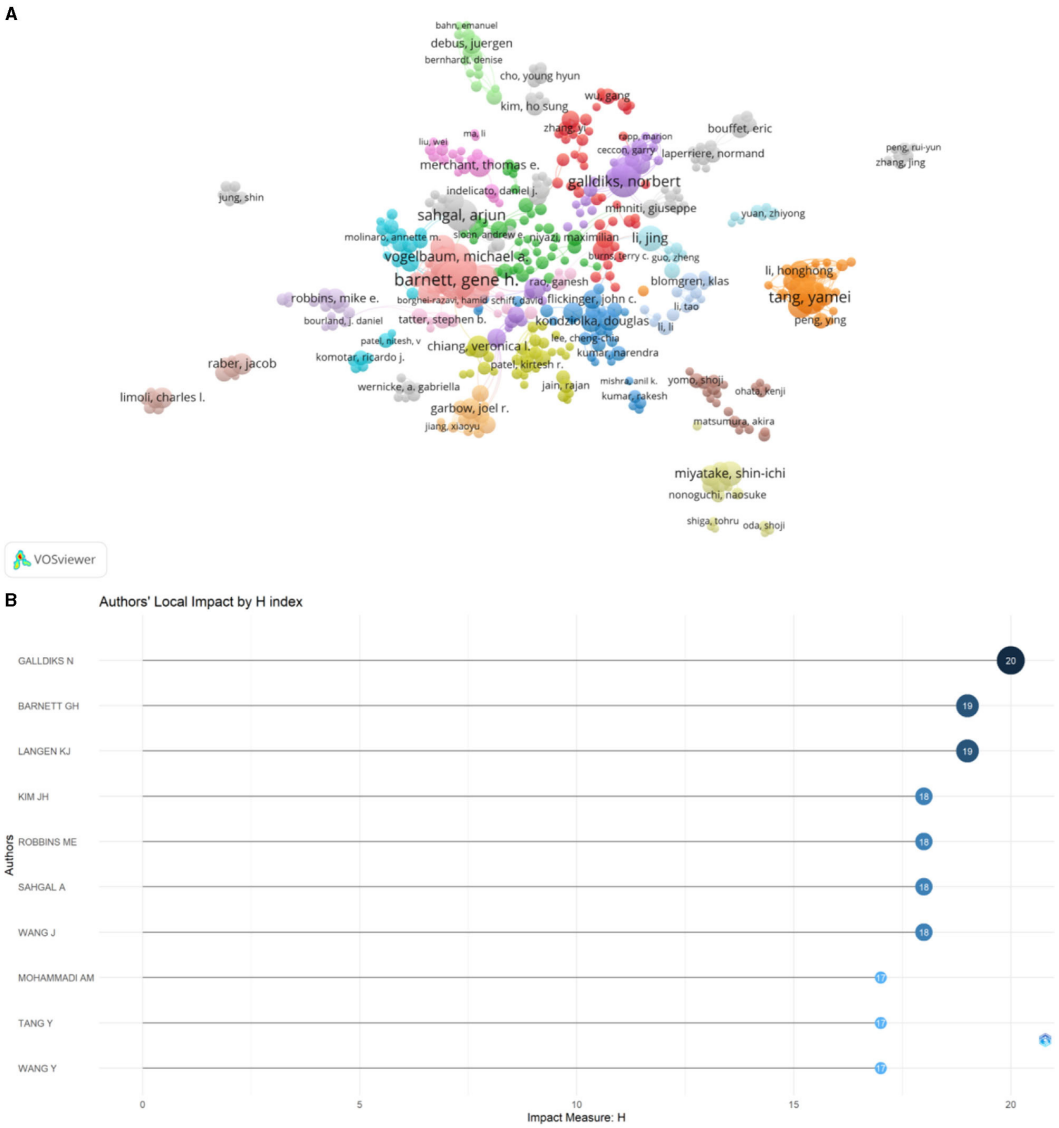
The bibliometric analysis of authors on RBI. **(A)** The network map of authors. **(B)** The top 10 authors of local impact.

A total of 21,529 authors contributed to radiation encephalopathy publications. The top 10 authors with the highest number of publications are listed in Table 2. The evaluation criteria for core authors in this field include the number of publications in the field, the number of citations in the field, and the H-index. Accordingly, Galldiks N, Barnett GH, Langen KJ, and Kim JH are known to be core authors in the field (Figure 6; Table 2).

**Table 2:** Top 10 publication authors and representative literature.

**Authors**	**Publications**	**Total citations**	**H_index**	**Representative literature**	**Journal**	**IF**	**Citations of literature**
Wang Y	50	1,119	17	Reversal of cerebral radiation necrosis with bevacizumab treatment in 17 Chinese patients	European journal of medical research	4.2	49
Wang J	44	768	18	Rapamycin increases collateral circulation in rodent brain after focal ischemia as detected by multiple modality dynamic imaging	Theranostics	12.4	27
Tang Y	40	815	17	Psychological disorders, cognitive dysfunction and quality of life in nasopharyngeal carcinoma patients with radiation-induced brain injury	PLoS ONE	3.7	72
Sahgal A	34	959	18	Prescription dose guideline based on physical criterion for multiple metastatic brain tumors treated with stereotactic radiosurgery	International journal of radiation oncology biology physics	7.0	29
Kim JH	33	1,156	18	Mechanisms of radiation-induced brain toxicity and implications for future clinical trials	Journal of neuro-oncology	3.9	104
Barnett GH	31	1,252	19	Laser interstitial thermal therapy for focal cerebral radiation necrosis: a case report and literature review	Stereotactic and functional neurosurgery	1.7	112
Galldiks N	26	1,282	20	The use of dynamic O-(2-18F-fluoroethyl)-L-tyrosine PET in the diagnosis of patients with progressive and recurrent glioma	Neuro-oncology	15.9	146
Langen KJ	25	1,296	19	Imaging of amino acid transport in brain tumors: positron emission tomography with O-(2-[18F]fluoroethyl)-L-tyrosine (FET)	Methods	4.8	66
Mohammadi AM	25	854	17	Laser interstitial thermal therapy in treatment of brain tumors—the NeuroBlate System	Expert review of medical devices	3.1	82
Robbins ME	22	1,829	18	The AT1 receptor antagonist, L-158,809, prevents or ameliorates fractionated whole-brain irradiation–induced cognitive impairment	International journal of radiation oncology biology physics	7.0	80

### 3.4 Analysis of journals

This study found that a total of 1,219 journals published articles on radiation encephalopathy, with a total of 142,473 citations. Among the top 10 journals in terms of publications, “Journal of Neuro-oncology” (IF = 3.9) is the top journal with 217 articles and the second highest number of citations (6,662). “International Journal of Radiation Oncology Biology Physics” (IF = 7.0) has 116 articles published in it and is the most referenced journal with 11,704 citations, making it the 2rd-most published journal. “Journal of Neurosurgery” is the third most referenced journal with 5,292 citations. The remaining seven journals in the top 10 have more than 60 articles each (Table 3).

**Table 3:** Ranking of top 10 cited journal in the field of RBI.

**Sources**	**Articles**	**Citation**	**IF**
Journal of neuro-oncology	217	6,662	3.9
International journal of radiation oncology biology physics	166	11,704	7.0
Journal of neurosurgery	126	5,292	4.1
World neurosurgery	87	1,120	2.0
American journal of neuroradiology	77	4,279	3.5
Neurosurgery	77	3,515	4.8
Radiation oncology	75	2,025	3.6
Frontiers in oncology	69	1,380	4.7
Neuro-oncology	64	3,521	15.9
Radiation research	62	1,754	3.4

**Figure 7:**
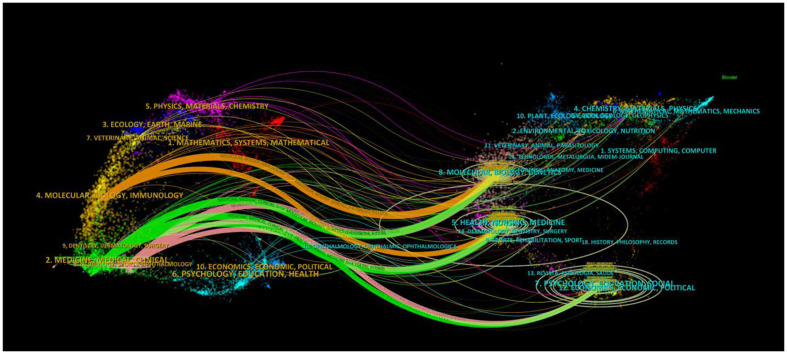
The dual map of journals on RBI (The number of publications and authors in RBI is indicated by the size of the ellipse. The ellipse's horizontal axis and vertical axis both display the number of writers and publications, respectively. The citation direction is from the cited journal on the right to the citing journal on the left, and the lines show citation links between journals).

The dual map was mainly used to explore the relationship between disciplines. The journal on the left cites the journal on the right. A citation connection was displayed in the curve style as a spline curve that ran from the citing journal's source to its target (21). Figure 7 depicts 2 primary colored citation curves. The yellow line is split into two branches, indicates that more material is cited in the “molecular biology” and “genetics” directions than in the “molecular biology” and “health nursing and medicine” directions. The green main line is split into three branches, suggesting that the literature in the fields of “pharmacology” and “clinical medicine”, respectively, cites “genetics and molecular biology”. Another field, “health and nursing” does the same with “molecular biology”. “health and nursing” also, respectively, cites “psychology, education, and social”.

### 3.5 Analysis of top 10 citations of included records

Of the 4,913 papers analyzed in this study, the top 10 publications ranked by citation are listed in Table 4. The most highly cited paper, published in the journal Science by Monje et al. (23), reported that neuroinflammation alone inhibits neurogenesis and that inflammatory blockade with indomethacin, a common non-steroidal anti-inflammatory drug, restores neurogenesis after endotoxin-induced inflammation and augments neurogenesis after cranial irradiation. This paper has received a total of 1,886 citations, with a normalized total of 26.24 citations, significantly higher than the second most highly cited paper. Eight of the top 10 records were published in journals with an IFQ1, including four top journals such as Science, Lancet, Journal of Clinical Oncology, and Nature Reviews Molecular Cell Biology. The topics covered included intratumoral heterogeneity in primary glioblastoma, metastatic melanoma, and cell types in the mouse cortex and changes in hippocampus neural stem cells, neuronal apoptosis, and radiation-induced cognitive impairments in Whole-brain radiation therapy. The most recent paper, published in 2019, has been cited 950 times.

**Table 4:** Ranking of top 10 cited publications in the field of RBI.

**References**	**Total citations**	**Total citations (TC)**	**Normalized TC**
Monje et al. (23) Science	Inflammatory blockade restores adult hippocampal neurogenesis	1,886	26.24
Chang et al. (24) Lancet Oncol	Neurocognition in patients with brain metastases treated with radiosurgery or radiosurgery plus whole-brain irradiation: a randomized controlled trial	1,777	21.13
Kuppermann et al. (25), Lancet	Identification of children at very low risk of clinically-important brain injuries after head trauma: a prospective cohort study	1,059	12.59
Singh et al. (26), Nat Rev Mol Cell Biol	Regulation of apoptosis in health and disease: the balancing act of BCL-2 family proteins	950	45.9
Brandsma et al. (27), Lancet Oncol	Clinical features, mechanisms, and management of pseudoprogression in malignant gliomas	799	12.97
Gondi et al. (28), J Clin Oncol	Preservation of memory with conformal avoidance of the hippocampal neural stem-cell compartment during whole-brain radiotherapy for brain metastases (RTOG 0933): a phase II multi-institutional trial	700	21.4
Zeng et al. (29), Int J Radiat Oncol Biol Phys	Anti-PD-1 blockade and stereotactic radiation produce long-term survival in mice with intracranial gliomas	641	15.57
Brandes et al. (30), J Clin Oncol	MGMT promoter methylation status can predict the incidence and outcome of pseudoprogression after concomitant radiochemotherapy in newly diagnosed glioblastoma patients	624	10.13
Mizumatsu et al. (31), Cancer Res	Extreme sensitivity of adult neurogenesis to low doses of X-irradiation	564	7.85
Barker et al. (32), Int J Radiat Oncol Biol Phys	Quantification of volumetric and geometric changes occurring during fractionated radiotherapy for head-and-neck cancer using an integrated CT/linear accelerator system	552	9.62

### 3.6 Analysis of co-citation references and citation burstness

**Figure 8:**
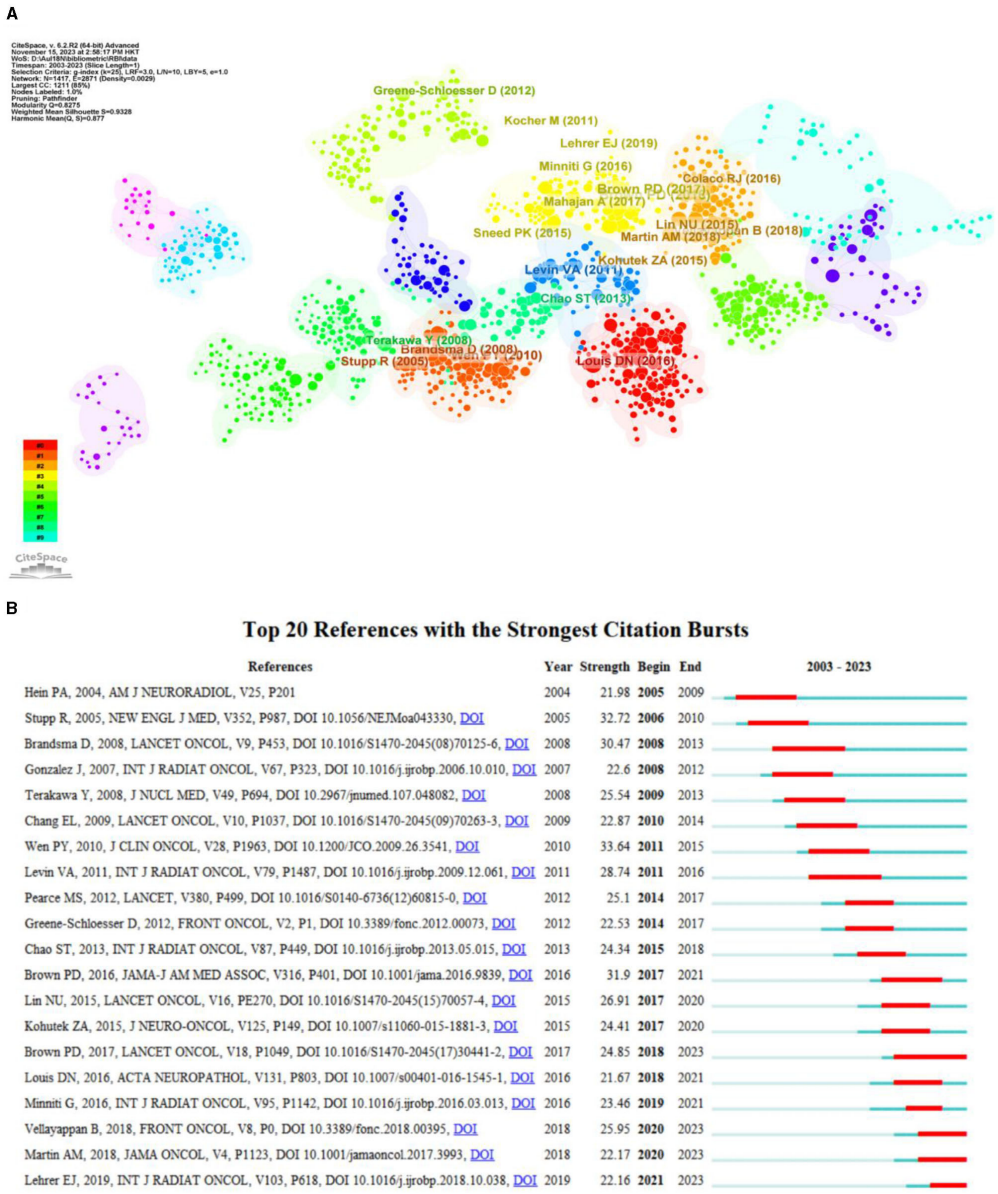
**(A)** The cluster view of co-citation references in the RBI research field. **(B)** The top 20 references with the strongest citation bursts.

In this study, CiteSpace was used to visualize the co-citation network of references, which were divided into 18 co-citation clusters (Figure 8A). The largest cluster was “radiation necrosis” (Cluster 0, *n* = 136), followed by “radiation injury” (Cluster 1, *n* = 121) and “brain metastases” (Cluster 2, *n* = 116). Citation burstness refers to references that receive a high level of attention from scholars in a specific field over a period of time. In CiteSpace, the minimum duration of burstness was set to 1 year for radiation encephalopathy, and 10 references with strong citation burstness were identified (Figure 8B). The strongest burstness (*n* = 33.64) among the top 10 references was observed for the paper entitled “Updated response assessment criteria for high-grade gliomas: response assessment in neuro-oncology working group” by Wen et al. (24), with citation burstness from 2011 to 2015. The remaining three references with the strongest burstness had values >30 and showed citation burstness from 2006 to 2021. Overall, the burstness strength of the top 10 references ranged from 21.67 to 33.64, while the duration of burstness ranged from 2 to 5 years.

### 3.7 Analysis of keywords

**Figure 9:**
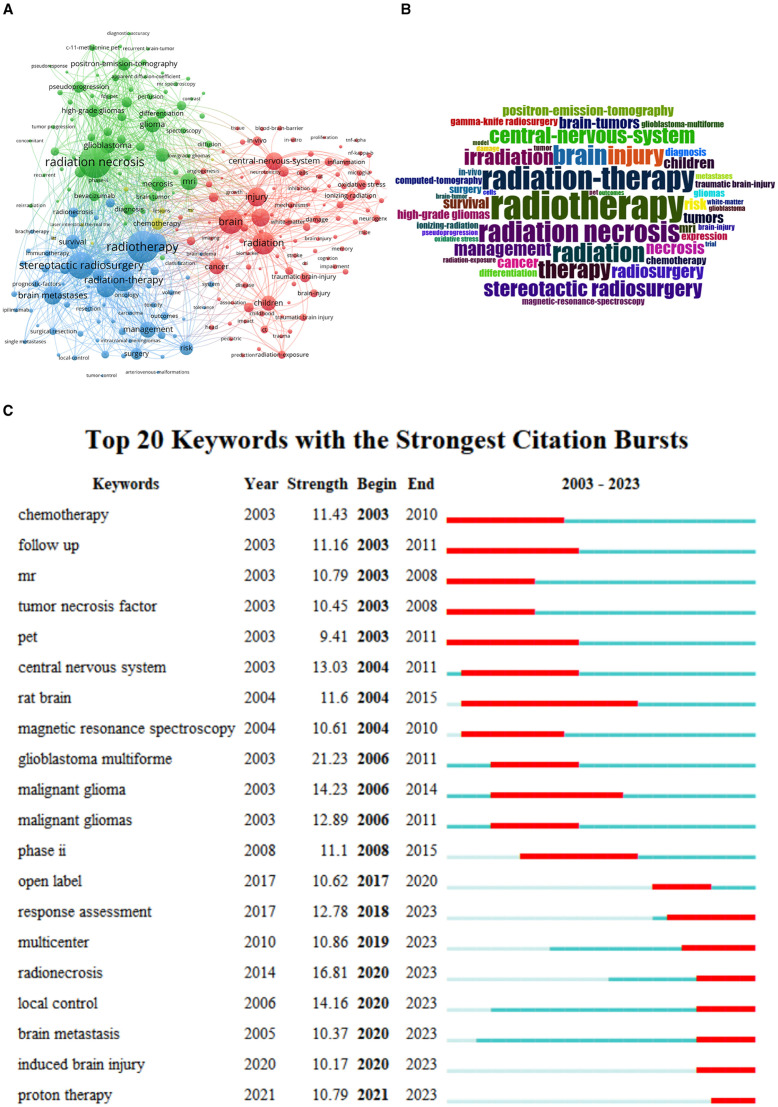
**(A)** The cluster view of high frequency keywords in the RBI research field. **(B)** The WordCloud of high-frequency keywords in the field of RBI. **(C)** The overlay visualization of the top 10 frequency keywords between 2003 and 2023.

From the 4,913 published records, a total of 8,231 keywords were extracted. The network map of the top 250 frequency keywords was clustered and formed 4 clusters (Figure 9A), and the biggest 3 nodes were “radiation,” “radiation necrosis,” and “radiation-therapy”. In the study on RBI, Figure 9B shows the emphasized terms, which include radiotherapy, radiation-therapy, radiation necrosis. Moreover, the overlay visualization of the top 10 burstness keywords between 2003 and 2023 is shown in Figure 9C, which are words that occur frequently within a specific time period. These burst keywords indicate the evolution of research hotspots and reveal current research trends and potential future trends. In this study, the burst keyword with the highest strength is “glioblastoma multiforme”. However, induced brain injury and proton therapy have been receiving increasing attention in recent years.

## 4 Discussion

Radiation therapy is regarded as a crucial treatment for brain cancers, and studies have demonstrated its effectiveness. Radiotherapy, however, entails a risk of neurologic harm, such as myelopathy, brachial plexopathy, localized brain necrosis, and cognition impairment related to cerebrovascular disease. Many individuals with brain malignancies continue to suffer radiological brain damage following radiation therapy, despite advancements in technology. Research has indicated that individuals who survive longer than a year following standard irradiation for malignant glioma have a 10% to 15% incidence of RBI (33). In patients with nasopharyngeal cancer treated with standard radiation treatment (<6,000 cGy) for 9 months to 16 years, the incidence of temporal lobe necrosis ranges from 1.6 to 22.0% (34). According to some research, healthy individuals who are exposed to low-dose radiation for an extended length of time may also suffer varying degrees of brain damage (35). Thus, there is a huge impact on healthcare and society from excessive radiation exposure and a high prevalence of RBI. It is important to include the indirect, administrative, and medical expenses associated with RBI. Patients with RBI benefit from prompt diagnosis and early management, but sufficient awareness of the condition still has to be raised. At the moment, RBI is becoming more and more well-known globally, and the amount of study on the subject is growing yearly. As a result, it is especially crucial to give a thorough summary of the most recent international trends in RBI research. This analysis identifies the main research hotspots and trends from 2003 to 2023 by analyzing the bibliometric features of the worldwide RBI field.

### 4.1 Principal results

The quantity of articles published annually and patterns found in the literature may serve as indicators of the study's development and research advancement. The publishing of research literature on RBI has trended upward globally over the past 20 years (Figure 3), with two peak periods. 2014 had the first peak of 285 publications, an increase of 54 over the previous year. The increase in publications in 2014 might be attributed to the conclusions of several previous research on radiation damage. In 2009, results from a clinical study by Chang et al. were published in a Lancet supplement (36). These results showed that patients who underwent a 4-month course of stereotactic radiosurgery (SRS) plus whole-brain radiation therapy (WBRT) were more likely than those who received SRS alone to suffer from severe deficits in learning and memory performance. By exposing mouse brains to radiation, Parihar and Limoli shown that radiation has a long-lasting detrimental effect on synaptic plasticity and dendritic complexity, which eventually leads to a decline in cognitive performance (37). The citation mutation analysis (Figure 8B) indicates that the aforementioned articles are among those with strong reference value and guidance in 2012–2017, offering the framework and direction for additional research. This conclusion is based on the perspectives of publication time, sustained citation time, and citation strength. Moreover, the rise in 2021 can be related to the numerous evaluations of RBI research that were published in 2016 (Figure 8B). The notable surge in publications during the last 12 years can be ascribed to multiple factors, such as the progress made in radiotherapy technology, the extended survival of numerous tumor patients, the increasing acknowledgment of the detrimental consequences of radiation, and the pressing requirement for remedial actions. Additionally, early detection of radiation damage is now feasible because to the use of innovative diagnostic methods including high-resolution magnetic resonance imaging (MRI) and DTI. This growth could have also been influenced by developments in the domains of immunology, neurology, microbiology, and oncology.

More than 40% of all RBI papers worldwide come from the United States, and the majority of the most prestigious and active research institutes are found in industrialized nations, which advances the field of RBI-related study. The way malignancies are treated in industrialized nations like the US might be connected to this trend. Radiotherapy is the main treatment for 50% of patients in North America with brain tumors (38). RBI are becoming more commonplace as a result of improved radiation therapy techniques and longer survival times. There is an increasing demand for RBI to be treated effectively. As a result, these nations or areas have made significant investments in this area of study, which has led to a rise in the quantity of publications. Meanwhile, the US collaborates with other nations the most frequently. While China, Japan, and Germany publish a lot of papers, their low total link intensity (TLI) suggests that they don't often work together worldwide (Figure 4B). Low TLI countries should improve international collaboration and exchange by forming cordial cooperative ties, particularly with colleagues who have achieved noteworthy breakthroughs in this area. Additionally, out of the 21,529 writers who have written in this topic, the top 200 come from collaborative networks that provide major contributions to the study, mostly from the nations and institutions indicated above.

**Figure 10:**
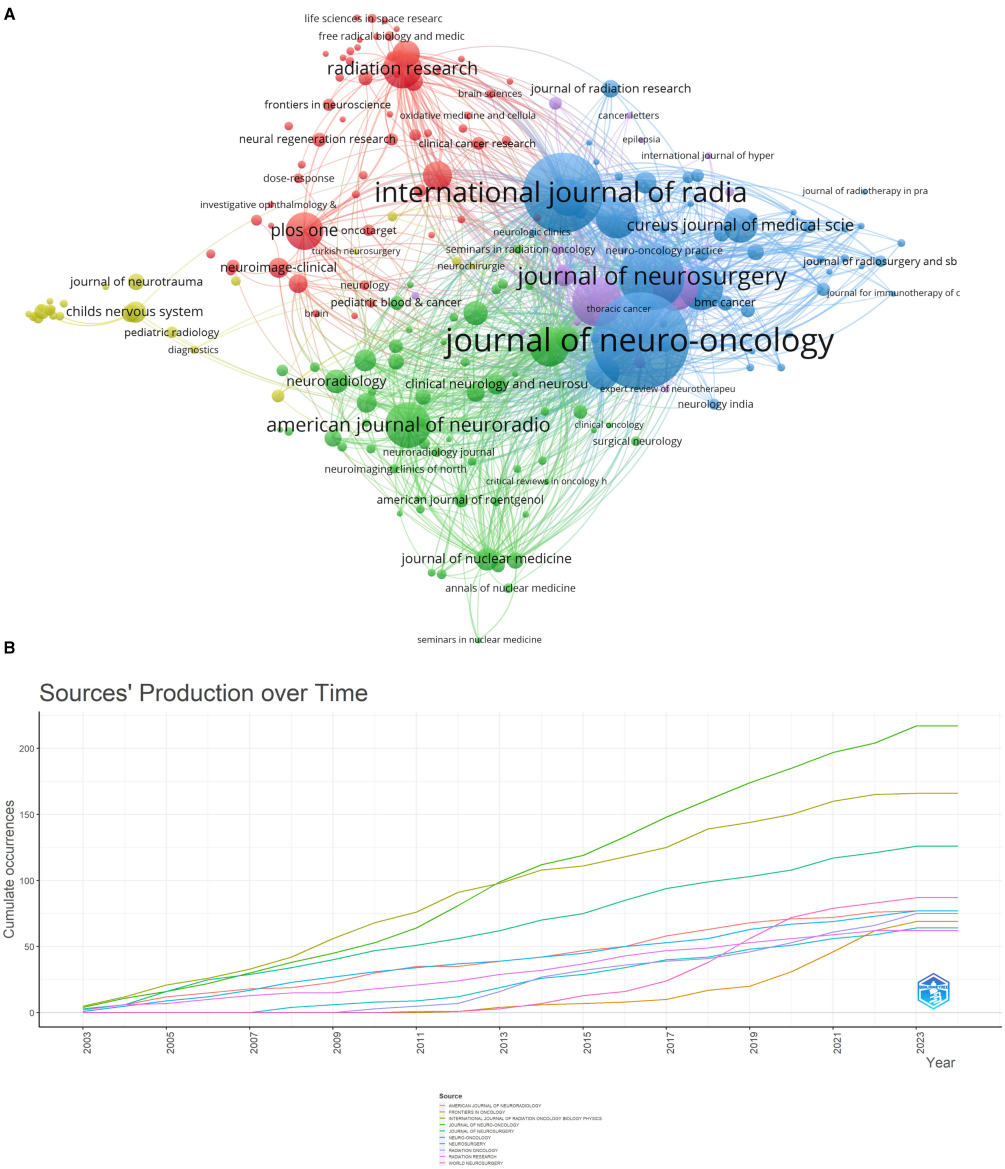
**(A)** The network map of Journals. **(B)** Cumulative publication trends of the top-10 most prolific journals (2003–2023).

The Journal of Neuro-Oncology is the journal that has published the most articles pertaining to RBI. Researchers may choose which journals to submit manuscripts to by having knowledge of popular journals; articles from highly cited journals can also be utilized as reliable sources of information (39). The Journal of Neuro-Oncology, the International Journal of Radiation Oncology Biology Physics, and the Journal of Radiology Oncology Biology Physics were the three publications that placed in the top 10 highly prolific and co-cited journals (Figure 10A; Table 3). Experts in the area heartily endorse all three publications. Given the renown of these publications in the disciplines of neurology and radiation oncology, it is likely that RBI is a widely discussed issue in neurology and radiology departments. Figure 10B shows that throughout the previous 20 years, there has been a consistent rise in the quantity of papers published in a number of these important journals. It suggests that one of the side effects of treating brain tumors that cannot be disregarded is RBI. Interestingly, a large number of RBI studies have been published in publications related to neuro-oncology surgery throughout the past 5 years. The advent of SRS in neuro-oncology surgery may be connected to this. For a number of brain tumors that were previously exclusively amenable to open neurosurgery, SRS provides a non-invasive and effective treatment modality. To properly locate intracranial target locations, the procedure was created by combining radiation treatment with stereotactic equipment. Meningiomas, gliomas, and brain metastases are among its indications (40, 41). Patients with brain tumors are living longer thanks to the growing safety and loosening of stereotactic radiology justifications, but there is no denying that the frequency of radiation-related problems has gone up. According to certain studies, 5–25% of patients with brain metastases who get SRS experience radionecrosis (42). Neurosurgeons, radiologists, and oncologists will collaborate more closely to better investigate RBI, as seen by the focus neurosurgical oncologists have started to give to the prevention and management of this kind of problem. The double-figure overlay shows the journal distribution and macro picture of the discipline's evolving research content. Molecular biology and clinical medicine are the two main fields associated with RBI, as seen in Figure 7. The visualization's five main routes demonstrate how RBI research is starting to adopt an interdisciplinary viewpoint as opposed to a disciplinary one. the thorough integration of molecular biology, pharmacology, nursing, and clinical medicine Early identification and intervention may be facilitated by the merging of clinical medicine, pharmacology, molecular biology, and nursing via thorough inquiry into the pathophysiology of radiation encephalopathy. The progress of molecular biology in various fields will be made possible by the advancement of biological experimentation techniques.

In general, high ranking co-citations often indicate the “knowledge base” of the field (39). For academics who wish to quickly learn about a certain area, these publications might serve as a foundation. Radiation-induced neuronal apoptosis and radiation-induced cognitive impairment were chosen as current cutting-edge subjects for our investigation, as can be shown in the Results section (Figure 8; Table 4). According to a research by Chang et al. (36), patients receiving SRS in addition to WBRT had a higher chance of experiencing a notable loss in memory and learning over the course of 4 months. An extensive overview of the inflammatory reactions and immunological alterations brought on by radiation therapy in the brain is given by Lumniczky et al. (43). The scientists came to the conclusion that complicated communication between different brain cells, such as neurons, microglia, astrocytes, and endothelial cells, as well as the peripheral immune system, is what causes radiation-induced neuroinflammation. They discovered a connection between neuroinflammation and the emergence of severe neurodegenerative illnesses and cognitive decline. In a study that was released in 2018, Vellayappan et al. (44) investigated the risk factors that lead to the development of radionecrosis in patients who had brain metastases, as well as methods for diagnosing and treating the condition. In comparison to single-fraction radiosurgery, the treatment of large brain metastases with multiple-fraction radiosurgery regimens may give a relative reduction in radionecrosis while preserving or increasing relative rates of 1-year Local control, according to the findings of a meta-analysis by Lehrer et al. (45). It is noteworthy that these two papers have the strongest citation bursts in the last 3 years (Figure 8B).

A total of 8,231 keywords were used in publications published on the topic of RBI research between 2003 and 2023. In bibliometrics, the keyword emergence analysis approach may be used to find terms that appear often throughout time and indicate the popularity of research in a certain field. The research focus on RBI was observed at various times over 20 years: “central nervous system” (2003–2006), “glioblastoma multiforme and malignant glioma” (2006–2011), and “radionecrosis” (2018–2023). The primary goal of early research on RBI was to accurately diagnose and distinguish it from other brain tumors. Wang's work showed that N-acetyl aspartic acid (NAA)/choline (Cho) was significantly lower in the RBI group and that (1) H-MRS in combination with diffusion tensor imaging (DTI) may detect RBI in patients undergoing therapy for nasopharyngeal cancer. The three most important terms with a strong breakout intensity over the previous 5 years were radionecrosis, local control (LC), and brain metastasis. This might also mean that, in order to enhance local tumor management and lower the risk of radionecrosis in patients with brain metastatic cancer, the present focus of RBI research is on developing new radiotherapy modalities and integrating pharmaceutical therapies. Patients with brain metastasis are frequently treated with SRS, which has a high LC rate. Brain radionecrosis (RN), the most frequent long-term adverse effect of SRS, is linked to a variety of neurological abnormalities in as many as one-third of patients (46, 47). High LC rates have led to the adoption of hypofractionated stereotactic radiotherapy (hFSRT) as an SRS substitute. Numerous retrospective investigations have documented an increase in the number of patients treated with SRS using doses of 24–35 Gy in 3–5 portions. An annual LC rate of 70–90% has been reported in a number of retrospective investigations, with an RN risk that varies from 2 to 15% (48, 49). RBI is mostly mediated by elevated levels of vascular endothelial growth factor (VEGF) expression following vascular damage. By blocking VEGF and affecting the vascular tissues surrounding the area of brain necrosis, bevacizumab reduces the symptoms of cerebral edema brought on by radiation-induced brain necrosis. Bevacizumab has been shown in several studies to be efficacious in treating symptoms resulting from brain necrosis. According to a research by Gabriella Wernicke et al. (50), patients with recurrent glioblastoma who received Cs-131 brachytherapy in addition to bevacizumab were able to retain LC and avoid RN.

### 4.2 Recommendations for future work

Thanks to advancements in therapy, patients with primary and metastatic brain cancers now have a significantly higher overall survival rate. However, this improvement comes with a variety of problems, including tumor recurrence and post-radiation treatment effect (PTRE), including radiation necrosis and pseudo-progression (51). RN has imaging characteristics that are comparable to tumor recurrence on T1-weighted, contrast-enhanced, T2-weighted, and fluid-attenuated inversion recovery (FLAIR) magnetic resonance imaging. In an effort to distinguish pseudo-progression from radiation necrosis and tumor recurrence, a variety of imaging techniques, including perfusion MRI, magnetic resonance spectroscopy (MRS), positron emission tomography (PET), single-photon emission spectroscopy (SPECT), and multimodal diagnostic approaches in imaging histology, have demonstrated encouraging results (52). Regretfully, there aren't many standards in the literature to figure out these results after therapy. Future studies are required to provide diagnostic imaging standards for the many imaging presentations of radiation necrosis, pseudo-progression, and tumor recurrence. There is evidence to suggest that limiting radiation doses to the temporal lobe and hippocampus is important to minimize the neurocognitive sequelae of radiation to the brain (53), but we currently lack enough information to determine the safe radiation tolerance of the various brain structures. To properly protect these areas using methods like intensity-modulated radiotherapy, it is imperative to have a better understanding of these structures' radiation tolerance and dose response. Likewise, it is crucial to assess dose-volume effects in these structures in more detail. Few therapies for RBI have demonstrated clear effectiveness, despite the fact that several have been suggested (54). Due to the intricate pathophysiology of radiation brain damage, neuroprotective therapies alone are currently restricted for RBI, despite the fact that they have been the focus of several trials with mediocre outcomes (55). Therefore, to avoid and minimize radiation-related morbidity as well as to enhance treatment approaches, a deeper comprehension of the cellular and molecular mechanisms underlying the development of radionecrosis is required.

### 4.3 Strengths and limitations

Indeed, to our knowledge, this is the first bibliometric evaluation of studies on RBI. We are able to fully comprehend the state of research on RBI using three visualization tools to locate hotspots, authors, nations, and institutional partnerships. This research does, however, have significant drawbacks. First, we only used data from the WoSCC and only included English-language articles to prevent selection bias. Despite this, our study's data collection is enough to give a broad picture of the state of the art in studies on RBI. Second, bias could still persist despite good normalization of the data since various expressions for the same author names or keywords were used. Thirdly, there may have been some logical inconsistencies between CiteSpace and VOSviewer on the same findings, and some information might have been overlooked because of software use, leading to mistakes in data analysis.

## 5 Conclusions

The WoSCC database returned a total of 4,913 research papers on RBI that were written and published between 2003 and 2023. Using R-bibliometrix, hybrid analysis, and visualization tools such as CiteSpace and VosViewer, the number of publications, significant institutions and countries, published journals, lead authors, and collaboration networks were thoroughly analyzed. By analyzing co-occurrence networks, researchers can learn about potential opportunities for collaboration with other institutions and researchers. The objective and comprehensive bibliometric analysis also identifies current research hotspots and potential future directions, providing a retrospective perspective on RBI and offering useful guidance to researchers choosing research topics. Future development directions include the integration of multi-omics approaches and novel imaging techniques to improve the diagnostic accuracy of RBI and the search for new therapeutic targets.

## Data availability statement

The original contributions presented in the study are included in the article/supplementary material, further inquiries can be directed to the corresponding author.

## Author contributions

BW: Writing—original draft. SL: Visualization, Writing—original draft. JianW: Data curation, Writing—original draft. JiayW: Writing—original draft, Software. WQ: Visualization, Writing- review & editing. HG: Writing—original draft, Writing—review & editing.
